# The KEAP1–NRF2 System in Cancer

**DOI:** 10.3389/fonc.2017.00085

**Published:** 2017-05-04

**Authors:** Keiko Taguchi, Masayuki Yamamoto

**Affiliations:** ^1^Department of Medical Biochemistry, Graduate School of Medicine, Tohoku University, Sendai, Japan

**Keywords:** NRF2, KEAP1, cancer, metabolic reprogramming, cancer therapy

## Abstract

Cancer cells first adapt to the microenvironment and then propagate. Mutations in tumor suppressor genes or oncogenes are frequently found in cancer cells. Comprehensive genomic analyses have identified somatic mutations and other alterations in the *KEAP1* or *NRF2* genes and in well-known tumor suppressor genes or oncogenes, such as *TP53, CDKN2A, PTEN*, and *PIK3CA*, in various types of cancer. Aberrant NRF2 activation in cancer cells occurs through somatic mutations in the *KEAP1* or *NRF2* gene as well as through other mechanisms that disrupt the binding of KEAP1 to NRF2. Unregulated NRF2 confers on cancer cells high-level resistance to anticancer drugs and reactive oxygen species (ROS) and directs cancer cells toward metabolic reprogramming. Therefore, NRF2 has been studied as a therapeutic target molecule in cancer. Two strategies have been used to target NRF2 via therapeutic drugs: inhibition of NRF2 and induction of NRF2. NRF2 inhibitors may be effective against NRF2-addicted cancer cells in which NRF2 is aberrantly activated. These inhibitors have not yet been established as NRF2-targeted anticancer drugs for the treatment of human cancers. Diagnosis of NRF2 activation could facilitate the use of NRF2 inhibitors for the treatment of patients with NRF2-addicted cancers. Conversely, NRF2 inducers have been used or are being developed for non-cancer diseases. In addition, NRF2 inducers may be useful for cancer chemotherapy in combination with conventional anticancer agents or even NRF2 inhibitors.

## Introduction

**Figure 1:**
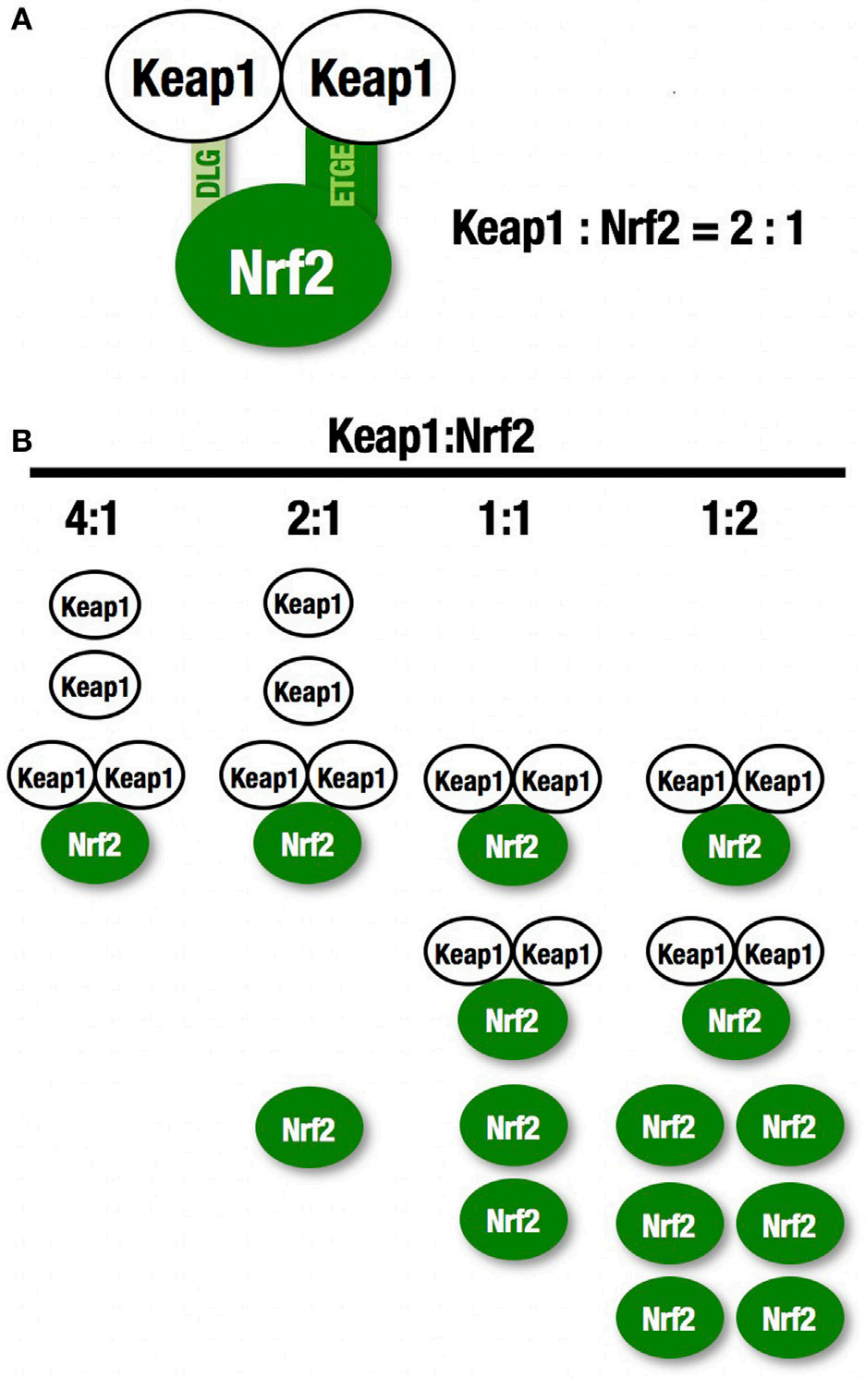
**KEAP1 and NRF2**. **(A)** Two KEAP1 molecules and one NRF2 form a trimer through the DC domain in KEAP1 and the DLG and ETGE motifs within the N-terminal Neh2 domain of NRF2. This two-site binding of NRF2 and KEAP1 has been shown to be the molecular basis of electrophile-induced NRF2 accumulation. **(B)** The ratio of KEAP1:NRF2 binding. KEAP1 is unable to bind a large amount of NRF2.

NF-E2-related factor 2 (NRF2) is a master regulator of numerous cytoprotective genes (1, 2). After translation, the NRF2 protein is rapidly degraded by the ubiquitin-proteasome system in the cytoplasm (3). Kelch-like ECH-associated protein 1 (KEAP1) is a component of the Cullin 3 (CUL3)-based E3 ubiquitin ligase complex and controls the stability and accumulation of NRF2. The KEAP1 DC domain directly binds NRF2 through DLG and ETGE motifs within the N-terminal Neh2 domain of NRF2 (4, 5) (Figure 1A). Since KEAP1 molecules form homodimers within cells, the stoichiometry of the KEAP1 homodimer and NRF2 is 1:1, and that of a single KEAP1 molecule and NRF2 is 2:1 (6) (Figure 1B). Thus, two KEAP1 molecules and one NRF2 molecule form a trimer, and the formation of this structure accelerates the ubiquitination of lysine residues in the NRF2 Neh2 domain, leading to proteasomal degradation of NRF2 (7–9). This two-site binding of NRF2 and KEAP1 has been shown to be the molecular basis of electrophile-induced NRF2 accumulation (10). Inactivation of KEAP1 strongly induces NRF2, and this phenomenon is often observed in cancer cells; cancer cells can thus acquire malignancy by perverting NRF2 activity. In this review, we will focus on the regulation of cellular NRF2 levels by KEAP1 and the perturbation of KEAP1 regulation in cancer cells.

## Mechanisms That Transmit Environmental Stresses to Gene Expression

In normal unstressed conditions, the cellular NRF2 level is very low (11), but it is dramatically increased upon exposure to electrophilic chemicals or reactive oxygen species (ROS) (1). Electrophiles modify reactive cysteine residues in KEAP1 (12). Murine KEAP1 possesses 25 cysteine residues, and these residues are categorized into several classes based on their reactivity to various electrophiles (13–15). For instance, cysteine 151 (C151) and C288 have been shown to sense distinct sets of electrophiles that are produced endogenously or exogenously (15–17). Oxidative modification of KEAP1 has also been shown to attenuate its binding to NRF2 or CUL3 (18, 19), although specific cysteine residues modified by ROS remain to be identified. These electrophilic and oxidative modifications inactivate KEAP1 and thereby stabilize NRF2. Therefore, the increase in NRF2 in response to electrophiles and ROS is not induction in a strict sense but rather is a mechanism referred to as derepression (from the rapid degradation-based repression). In this paper, we use both derepression and induction to describe this phenomenon, i.e., the increase in NRF2.

**Figure 2:**
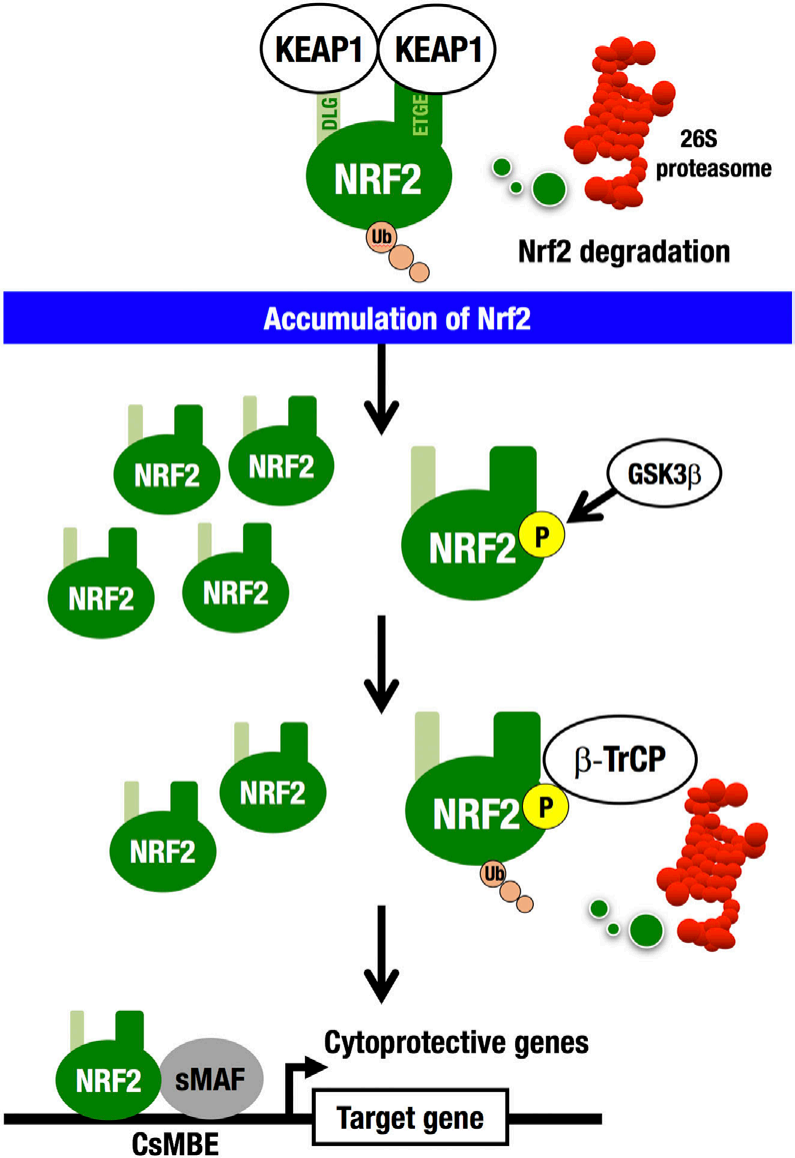
**KEAP1–NRF2 system**. KEAP1-CUL3 directly binds NRF2, which is then rapidly degraded by the 26S proteasome in the cytoplasm. NRF2 that escapes KEAP1 trapping is stabilized and accumulates in the nucleus. GSK3 phosphorylates the Neh6 domain of NRF2. Phosphorylated NRF2 binds β-TrCP-CUL1 and is then degraded by the 26S proteasome in the nucleus. NRF2 forms a heterodimer with sMAF and binds CNC-sMAF-binding elements (CsMBE), including the consensus ARE/EpRE sequence, TGACNNNGC. NRF2 upregulates cytoprotective genes encoding antioxidant enzymes and detoxifying enzymes.

For example, an electrophile, such as *tert*-butyl hydroquinone (tBHQ), reacts with reactive cysteine residues in KEAP1 to activate NRF2 (20). Importantly, binding of tBHQ to KEAP1 does not disrupt the binding of NRF2 to KEAP1 (21), demonstrating that simple dissociation of NRF2 from the KEAP1 homodimer cannot explain the electrophile-mediated induction of NRF2 accumulation within cells. Instead, the evidence indicates that since electrophilic modification inactivates KEAP1, newly synthesized NRF2 is able to escape KEAP1 trapping in this situation, so that the newly made NRF2 is stabilized and accumulates. KEAP1 is primarily localized to the perinuclear cytoplasm (22), loosely connected with the actin cytoskeleton (23), and serves to maintain the correct levels of NRF2 (6). KEAP1 forms an E3 ubiquitin ligase complex with CUL3, acting as a substrate recognition/binding subunit. The KEAP1-based ubiquitin ligase complex ubiquitinates NRF2, subjecting NRF2 to rapid proteasomal degradation in the cytoplasm (3) (Figure 2).

While this is the major pathway for the cellular response to NRF2 induction (derepression), there exists a second NRF2 degradation pathway within the nucleus. β-TrCP (β-transducin repeat-containing protein) forms an E3 ubiquitin ligase complex with CUL1 and ubiquitinates NRF2. β-TrCP serves as a substrate recognition/binding subunit that recognizes phosphorylated NRF2 (24). In the nucleus, the serine residues in the Neh6 domain of NRF2 are phosphorylated by glycogen synthase kinase 3 (GSK3), which is a downstream effector of the phosphoinositide 3-kinase (PI3K)–AKT pathway (25), and phosphorylated NRF2 is captured by β-TrCP, ubiquitinated, and subjected to proteasomal degradation.

Interestingly, in an experiment using mouse liver, deletion of the *PTEN* gene and subsequent activation of the PI3K–AKT pathway was insufficient to activate NRF2. Conversely, deletion of the *KEAP1* gene significantly activated NRF2. Simultaneous deletion of the *PTEN* and *KEAP1* genes activated NRF2 much more strongly than the *KEAP1* single deletion (26). Thus, NRF2 degradation occurs via two pathways (27); the major pathway is localized in the cytoplasm and governed by KEAP1, while the secondary pathway is in the nucleus and governed by β-TrCP. These observations support the notion that cellular NRF2 levels are strictly regulated by two pathways through the protein degradation-repression mechanism: derepression from the KEAP1-based repression causes a rapid increase in NRF2 activity and induction of cellular defense mechanisms against electrophilic and oxidative insults, while β-TrCP-based NRF2 degradation inhibits unnecessary NRF2 over-induction caused by KEAP1 inactivation.

It has been reported that there is a link between NRF2 activity and miRNAs that appears to be relevant to disease (28). For example, in breast cancer, miR-28 regulates NRF2 expression through a KEAP1-independent mechanism (29). miR-200a regulates NRF2 activation by targeting *KEAP1* mRNA in breast cancer cells (30). On the other hand, NRF2 regulates miR-1 and miR-206 to direct carbon flux toward the pentose phosphate pathway (PPP) and tricarboxylic acid (TCA) cycle, which reprograms glucose metabolism (31) (see the section on metabolic reprogramming in cancer).

## NRF2 Target Genes

Stabilized NRF2 translocates into the nucleus and forms a heterodimer with a small MAF (sMAF) transcription factor (1). The NRF2-sMAF heterodimer binds to antioxidant-responsive element (ARE) (32) or electrophile-responsive element (EpRE) (33) and induces transcription of numerous cytoprotective genes. The consensus ARE/EpRE sequence is TGACNNNGC (34). This sequence is highly similar to the consensus-binding sequence for the erythroid transcription factor NF-E2 (35), which is composed of a p45 subunit and sMAF. Historically, the NRF2-sMAF-mediated regulation of cytoprotective gene expression via ARE/EpRE was identified based on this similarity (1), as both p45 and NRF2 belong to a small transcription factor family named the Cap‘n’collar (CNC) family (36).

Recently, an extensive genome-wide analysis of the NRF2-sMAFF-binding sequence (i.e., the ARE/EpRE) and the MAF homodimer-binding sequence (the MAF responsive element or MARE) was conducted, and the differences between these elements were clarified (37). As a result, it was proposed that ARE, EpRE, and the NF-E2 binding sequence be collectively named CNC-sMAF-binding elements (CsMBE).

Recent chromatin immunoprecipitation-deep sequencing (ChIP-Seq) analyses have revealed target genes of the NRF2-sMAF heterodimer (34, 38–40). NRF2-sMAF appears to globally regulate cytoprotective and metabolic networks. One group of important NRF2-sMAF target genes encodes antioxidative enzymes and detoxifying enzymes. NAD(P)H:quinone oxidoreductase 1 (*Nqo1*) is representative of this group of genes and is widely used to evaluate NRF2 activity. In addition, NRF2-sMAF regulates genes encoding PPP enzymes, ABC pumps, and some heme-metabolizing enzymes.

## Aberrant NRF2 Activation in Cancer

**Figure 3:**
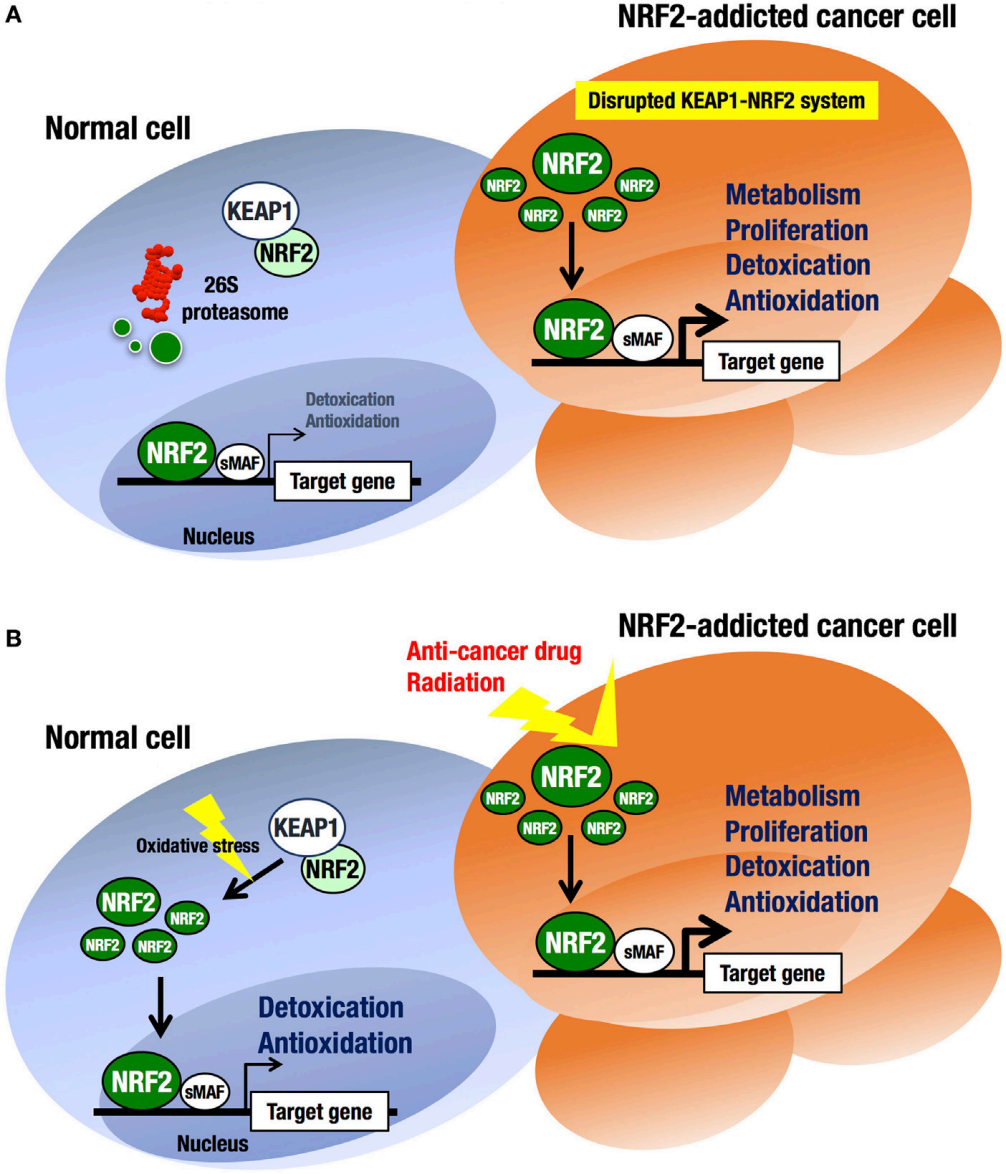
**Difference in NRF2 activation between normal cells and NRF2-addicted cancer cells**. **(A)** Unstressed condition. The intracellular NRF2 level is very low in normal cells. In contrast, constitutive NRF2 activation in cancer cells accelerates proliferation and metabolism. **(B)** Oxidative stress-exposed condition. In normal cells, the cellular NRF2 level is temporarily increased upon exposure to toxic (often electrophilic) chemicals and ROS. In contrast, constitutive NRF2 activation confers resistance on cancer cells to anticancer drugs and radiation.

An intriguing finding in human biology and pathology related to the KEAP1–NRF2 regulatory system is that cancer cells occasionally acquire aberrant NRF2 activation (41–43) (Figure 3A). At first, this observation was confusing, as the KEAP1–NRF2 system has been shown to contribute to cancer chemoprevention (44). However, previous observations that cancer cells often acquire strong antioxidative and drug-metabolizing activities explain cancer cell acquirement of malignancy and cytoprotective activity (45, 46).

At least four pathways have been reported to be involved in NRF2 activation in cancer cells (17). First, somatic mutations within the *NRF2, KEAP1*, or *CUL3* genes (41, 47–49) have been shown to cause aberrant NRF2 activation in cancer cells. Second, epigenetic silencing of the *KEAP1* gene has also been found to cause KEAP1 downregulation and NRF2 upregulation (50). Third, the accumulation of KEAP1 interacting proteins, such as p62/Sqstm1 (51) and p21 (52), has been found to block NRF2 binding to KEAP1, leading to NRF2 accumulation. Fourth, cysteine modification by oncometabolites such as fumarate affects KEAP1 activity and leads to NRF2 accumulation (53, 54). All these molecular events result in disrupted binding of KEAP1 to NRF2, causing aberrant accumulation of NRF2 in cancer cells. Unregulated NRF2 activates the target genes responsible for cytoprotection, conferring chemo- and radio-resistance on cancer cells (44) (Figure 3B).

The A549 cell line was derived from an adenocarcinoma of the human alveolar basal epithelium and is a typical cancer cell line that exhibits aberrantly active NRF2. There are two mechanisms by which A549 cells acquire constitutive NRF2 activation: one is a somatic mutation of the *KEAP1* gene at G333C (47, 55), and the other is epigenetic alteration by methylation in the *KEAP1* promoter (50). NRF2 is a key molecule that controls proliferation in NRF2-addicted cancer cells, such as A549 cells (26) (Table 1).

**Table 1:** **NRF2-addicted cancer cell lines**.

Cell line	Cancer types	Causes for NRF2 activation	Reference
**Lung**			
A549	Adenocarcinoma	*KEAP1* G333C (homo)	(47, 55)
		*KEAP1* promoter methylation	(50)
H838		*KEAP1* 443 frameshift (homo)	(47)
H1395		*KEAP1* G350S (hetero)	
H1993			
H1435		*KEAP1* L413R (homo)	
H460	Large cell carcinoma	*KEAP1* D236H (homo)	
**Esophagus**			
KYSE70	Squamous cell carcinoma	*NRF2* W24C (homo)	(56)
KYSE110		*NRF2* E82D (hetero)	
KYSE180		*NRF2* D77V (homo)	
**Kidney**			
Caki-2	Clear cell carcinoma	p62 accumulation	(57)
		KEAP1 silencing?	
UMRC-2		p62 accumulation	
SK-RC-20	Carcinoma	p62 accumulation	
UMRC-6		p62 accumulation	
SLR21		KEAP1 silencing?	
A498		n.d.	(58)
**Pancreas**			
AsPC-1	Adenocarcinoma	n.d.	(59)
Colo-357		n.d.	(60)
Suit-2		KEAP1 silencing?	(61)
**Prostate**			
DU145	Carcinoma	*KEAP1* promoter methylation	(31)
**Liver**			
JHH-5	Hepatocellular carcinoma	n.d.	(62)
Huh1		Phosphorylated p62 accumulation	

*The number in the causes for NRF2 activation column indicates the position of the mutated amino acid. Frameshift generates stop codon. homo, homozygous (2 mutant alleles); hetero, heterozygous (1 mutant allele); n.d., not yet determined*.

**Figure 4:**
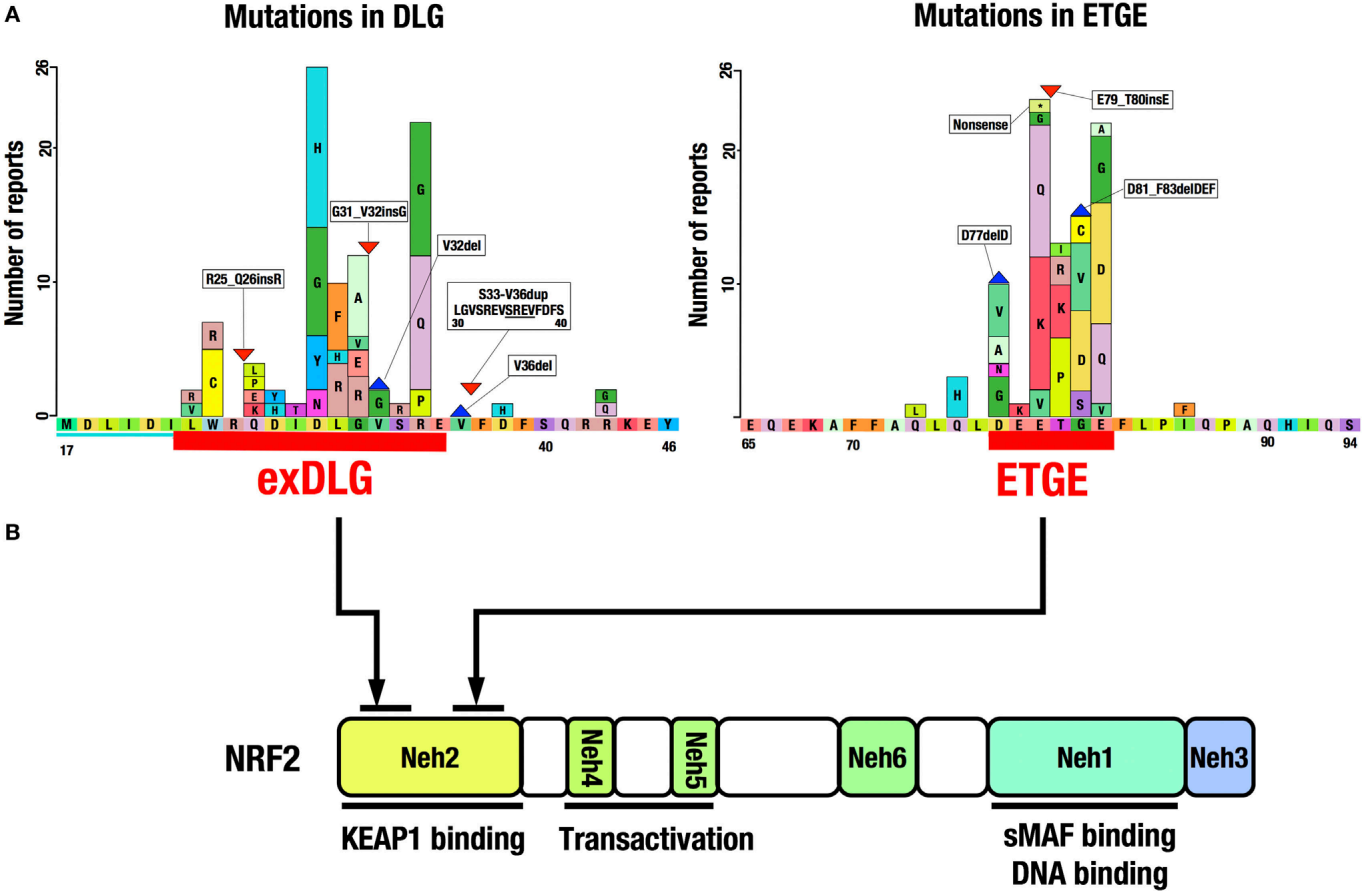
**Distribution of somatic mutations in the *NRF2* gene in cancers**. **(A)** *NRF2* mutations in the exDLG and ETGE motifs. Red lines indicate the DLG motif and the ETGE motif. Please see Ref. (10). **(B)** The domain structure of the NRF2 protein. *NRF2* mutations are exclusively found in the DLG and ETGE motifs responsible for binding to KEAP1.

A catalog of somatic mutations in cancer (COSMIC; http://cancer.sanger.ac.uk/cosmic) reveals 274 coding mutations in the *KEAP1* gene and 389 in the *NRF2* gene in cancers from various tissues (Figure 4A). Mutations in *KEAP1* or *NRF2* were found in approximately 0.9% of all cancer samples examined in studies published in COSMIC 2016. Interestingly, the *NRF2* mutations are exclusively found in the DLG and ETGE motifs responsible for binding to KEAP1 (Figure 4B). Cancer cells that harbor a somatic mutation in these motifs of the *NRF2* gene lose the KEAP1–NRF2 interaction and the subsequent constitutive repression of NRF2 activity during unstressed conditions.

*KEAP1* mutations, however, are widely distributed in the *KEAP1* gene and are found in virtually all domains of the protein. Somatic mutations in the *KEAP1* gene, similar to those in the *NRF2* gene, affect protein–protein interactions, i.e., the binding of NRF2 to KEAP1. Genomic alterations in various cancer-related genes have been comprehensively characterized in squamous cell lung cancers (63). The most frequently mutated gene was *TP53*, with mutations found in 81% of the analyzed samples. While *KEAP1* (12%) and *NRF2* (15%) mutations were found in a much lower percentage of samples, gene mutations related to the KEAP1–NRF2 system, including *CUL3* (7%) and *PTEN* (8%), were more frequent than those in the second most frequently mutated gene *CDKN2A* (15%).

Notably, mutations in *KEAP1, NRF2*, and *PTEN* are mutually exclusive and are seldom found in the same cancer cell (63). Therefore, correlations among mutations in KEAP1–NRF2 system genes and other well-known tumor suppressor genes or oncogenes are intriguing but have yet to be precisely studied. It was recently reported that mutations in either *KEAP1* or *NRF2* were found in approximately 14% of hepatocellular carcinoma (HCC) cases (64). This observation indicates that the frequency of mutations in *KEAP1* or *NRF2* depends on the cancer type and origin.

## Metabolic Reprogramming in Cancer

NRF2 target gene products are involved in cytoprotection, and typical examples include detoxifying enzymes and antioxidant enzymes. A ChIP-Seq analysis of NRF2 and MAFG target genes in A549 cells identified novel NRF2 target genes; for example, those encoding metabolic enzymes (26). In fact, NRF2 regulates the expression of genes encoding PPP enzymes and glutaminolysis-related enzymes. Previous studies have shown that the expression of malic enzyme 1, glucose-6-phosphate dehydrogenase (G6PD), and phosphogluconate dehydrogenase is correlated with NRF2 (65, 66). However, the functional implications of the induction of these enzymes in cancer cells are not clearly understood. Notably, siRNA-mediated *NRF2* knockdown in A549 cells that are addicted to NRF2 significantly decreases the proliferative ability of these cells (26). This observation suggests that NRF2 plays an important role in cancer cell proliferation. Based on this observation, we have proposed that NRF2 directs the metabolic reprogramming of cancer cells, a notion that has been supported by recent studies.

Both amplification of the *p62* gene and aberrant accumulation of phosphorylated p62 protein have been implicated in the acceleration of tumor development. Phosphorylation of p62 at S349 activates NRF2 and directs glucose metabolism to the glucuronate pathway and glutamine metabolism to glutathione synthesis (67). These changes make HCC cells resistant to anticancer drugs and enhance their proliferation ability. Phosphorylated p62 accumulates in tumor regions positive for hepatitis C virus (HCV). Similarly, hepatitis B virus (HBV) also stimulates NRF2 activation and upregulates G6PD, the first and rate-limiting enzyme of the PPP (68). NRF2 also reportedly regulates miR-1 and miR-206 to direct carbon flux toward the PPP and TCA cycle, and NRF2 also regulates the reprogramming of glucose metabolism in cancer cells (31).

## KEAP1–NRF2 System as a Therapeutic Target

**Figure 5:**
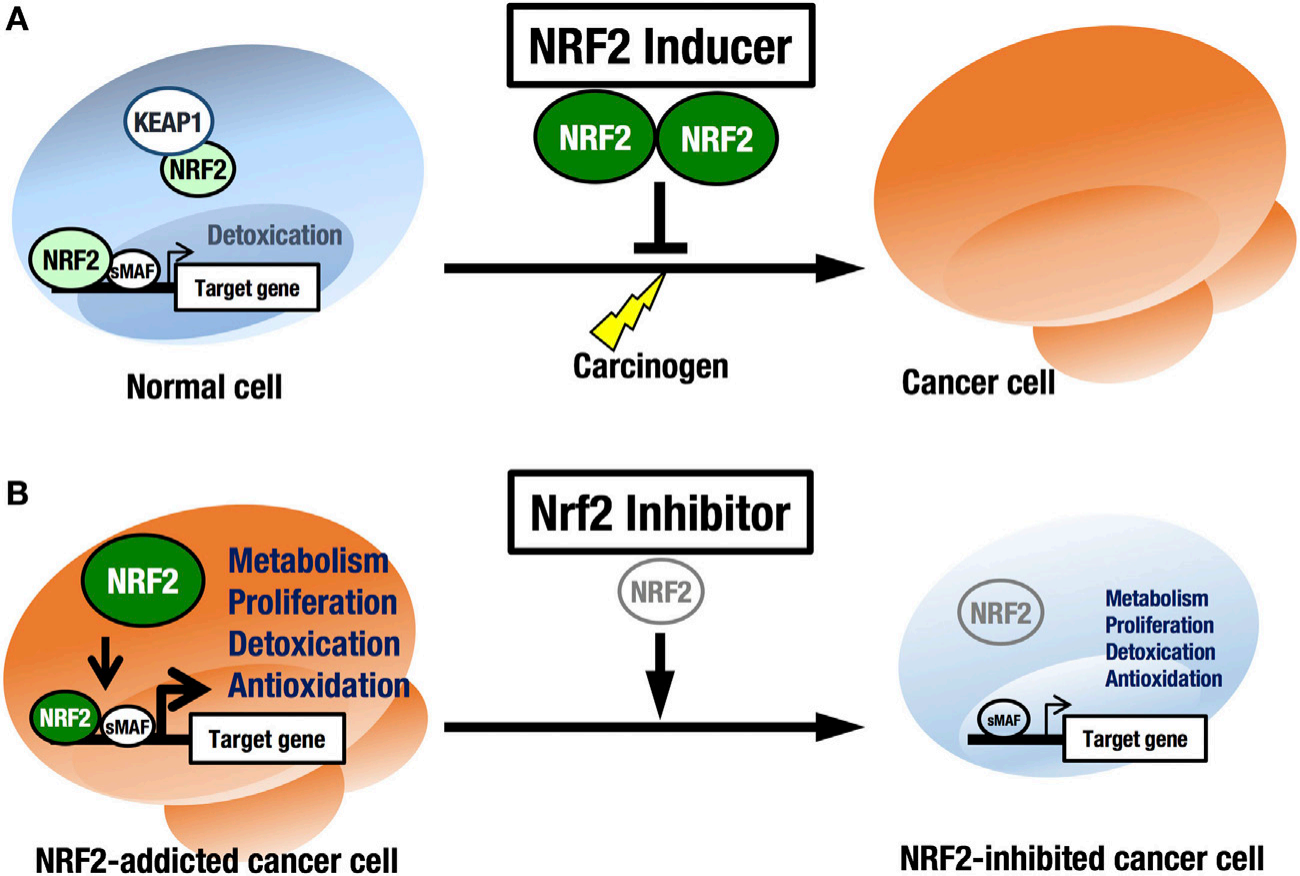
**Two strategies for cancer therapy focused on NRF2**. **(A)** Chemoprevention against carcinogens by NRF2 inducers in normal cells. **(B)** Anticancer therapy against NRF2-addicted cancer cells by NRF2 inhibitors.

NRF2 is an attractive molecule as a therapeutic target in cancer. There are two main strategies used to target NRF2 by therapeutic drug treatment: one is NRF2 inhibition, and the other is NRF2 induction. NRF2 inducers have been shown to accelerate the detoxification of carcinogens (often electrophiles) from the environment and protect the body from chemical carcinogenesis (Figure 5A). Of note, the NRF2 inducer dimethyl fumarate has been approved by the FDA for multiple sclerosis treatment, and bardoxolone methyl (CDDO-Me or RTA 402) is now in phase II clinical trials for pulmonary hypertension and chronic kidney diseases. Some phytochemicals, such as sulforaphane from broccoli sprouts (69), curcumin from turmeric (70), or carnosic acid from rosemary (71), also activate NRF2. These chemicals have been used as dietary supplements (72).

As described in the previous section, the chemicals described above act to modify KEAP1 cysteine residues (the cysteine code). Therefore, concerns remain regarding glutathione depletion or redox side effects. An alternative approach for the development of NRF2 inducers is the use of chemicals that disrupt the KEAP1–NRF2 interface, especially chemicals that target a pocket that resides in the bottom of the KEAP1 DC domain (10, 67).

In this regard, it should be noted that there are many cancers in a variety of tissues that show intrinsically high NRF2 activity. We refer to these cancers as NRF2-addicted cancers, as NRF2 provides cytoprotection to these cancer cells by activating detoxifying and antioxidative enzymes and through metabolic reprogramming. For these cancers, NRF2 inhibitors may show therapeutic effects (73) (Figure 5B).

## NRF2 Inhibitors

Brusatol is a type of degraded diterpenoid isolated from the *Brucea javanica* plant (74). Brusatol has been used in traditional Chinese medicine to treat a variety of ailments, including cancer, amoebic dysentery, and malaria. Brusatol inhibits DNA, RNA, and protein synthesis (75, 76), and inhibition of overall protein synthesis by brusatol has been observed in many types of cancer cells (74, 76, 77). Brusatol has recently been reported to act as an NRF2 inhibitor (78), inhibiting NRF2 in all cell lines tested, regardless of whether the *KEAP1* or *NRF2* genes were mutated. A subsequent paper showed that brusatol induces its potent cytotoxic effects in a manner independent of KEAP1–NRF2 activity and with a profile similar to a protein translation inhibitor. Therefore, brusatol does not specifically inhibit NRF2 but rather functions as a global protein synthesis inhibitor (79).

ML385, a thiazole-indoline compound, is a probe molecule that specifically binds to Neh1, the DNA, and sMAF-binding domain in NRF2, and inhibits the expression of downstream target genes (80). Combined with doxorubicin or Taxol, ML385 substantially enhances cytotoxicity in non-small cell lung cancer (NSCLC) cells compared to single agents. ML385 shows specificity and selectivity for NRF2-addicted NSCLC cells with *KEAP1* mutations, such as A549 and H460 cells. In preclinical models of NRF2-addicted NSCLC, ML385 shows significant antitumor activity in combination with carboplatin.

Febrifugine is the bioactive component of the traditional Chinese medical herb *Dichroa febrifuga* (81). The febrifugine derivative halofuginone has been optimized to be less toxic than febrifugine. Halofuginone is a specific inhibitor of collagen type-I synthesis (82) and of prolyl-tRNA synthetase (83). Febrifugine derivatives have been used as treatments for cancer, malaria, fibrosis, and inflammatory diseases. Halofuginone has been tested in phase 2 clinical trials for cancer (84) and fibrotic diseases (85) including Duchenne muscular dystrophy (86). Though HT-100, an oral analog of halofuginone, was studied in a phase 2 clinical trial for bladder cancer treatment, development of the compound was discontinued. Quite recently, halofuginone was found to inhibit NRF2 (87).

In NRF2-addicted cancer cells, halofuginone decreases NRF2 protein synthesis by inhibiting prolyl-tRNA synthetase. This inhibition is rescued by the addition of proline, supporting the hypothesis that halofuginone inhibits NRF2 by affecting the prolyl-tRNA synthetase. Halofuginone treatment of NRF2-addicted cancer cells, such as lung cancer-derived A549 cells or esophagus cancer-derived KYSE70 cells, attenuates proliferation *in vitro*. Co-treatment with halofuginone also enhances the effects of conventional anticancer drugs, such as cisplatin or doxorubicin, in a xenograft tumor model. While halofuginone is not a specific NRF2 inhibitor, it exerts a strong inhibitory effect on NRF2. This may be because NRF2 is a rapidly turned-over protein with a half-life of less than 20 min, and NRF2 synthesis is somehow linked to the amino acid starvation machinery. Thus, halofuginone could serve as a chemosensitizer in the treatment of various NRF2-addicted cancers.

Another challenge related to NRF2 inhibition is the inhibition of the protein–protein interaction between phosphorylated p62 and KEAP1. In many cases of HCC, phosphorylated p62 accumulates and inhibits KEAP1 activity by interacting with the NRF2-binding pocket of KEAP1. For example, Huh1 cells express a high level of phosphorylated p62 (64). Therefore, a specific inhibitor of the interaction between phosphorylated p62 and KEAP1 would enable KEAP1 to bind and rapidly degrade NRF2; such a drug would be expected to function as an anticancer drug, particularly for cancers such as HCC, in which phosphorylated p62 accumulates and inhibits KEAP1 activity and thereby increases NRF2 activity. K67 (*N*-[2-acetonyl-4-(4-ethoxybenzenesulfonylamino)naphthalene-1-yl]-4-ethoxybenzenesulfonamide) is an inhibitor of the phosphorylated p62–KEAP1 interaction that reduces the NRF2 level in HCC (67). K67 suppresses the proliferation of HCC cells and accelerates the effects of anticancer agents. K67 could also make cancer cells less resistant to anticancer drugs, especially in HCV-positive HCC patients.

Other chemicals have also been reported as NRF2 inhibitors, although the molecular mechanisms through which these chemicals inhibit NRF2 have not been well elucidated. The coffee alkaloid trigonelline suppresses proteasomal activity in pancreatic cancer cells following NRF2 inhibition (60). Chrysin (5,7-dihydroxyflavone) and apigenin (4′,5,7-trihydroxyflavone) sensitize doxorubicin-resistant human liver cancer-derived Bel-7402 cells to doxorubicin, which is associated with the downregulation of NRF2 (88). Luteolin (3′,4′,5,7-tetrahydroxyflavone) reduces NRF2 expression at both the mRNA and protein levels in NSCLC A549 cells (89).

## NRF2 Inducers

Aflatoxin B_1_ (AFB_1_) is a carcinogen contaminant in food. Exposure to AFB_1_ is one of the causes of HCC in Asia and Africa. While AFB_1_-driven HCC is experimentally reproducible in rats, it cannot be reproduced in mice. Mice are innately resistant to AFB_1_, because they express high levels of glutathione S-transferases (GSTs), which play an important role in AFB_1_ detoxification. CDDO-Im (1-[2-cyano-3-,12-dioxooleana-1,9(11)-dien-28-oyl] imidazole) is a synthetic oleanane triterpenoid and a powerful NRF2 inducer. CDDO-Im suppresses AFB_1_-induced toxicity and preneoplastic lesion formation (GST-P-positive foci) and completely protects rats against AFB_1_-induced HCC (90). With CDDO-Im treatment, the integrated level of urinary AFB_1_-N^7^-guanine is significantly reduced and aflatoxin-N-acetylcysteine, a detoxification product, is consistently elevated after the first AFB_1_ dose. In AFB_1_-treated rats, the hepatic burden of GST-P-positive foci increases, but the foci largely disappear after CDDO-Im intervention. The toxicogenomic RNA expression signature characteristic of AFB_1_ is absent in rats dosed with AFB_1_ in combination with CDDO-Im. The remarkable efficacy of CDDO-Im as an anticarcinogen is observed even in the presence of a significant aflatoxin adduct burden.

In this regard, GST-P has also been used as a marker of DEN (diethylnitrosamine)-induced hepatic micronodules in the Solt–Farber-resistant hepatocyte experiments (45). In the hepatic micronodules, GST-P appears to protect cancer cells from anticancer chemotherapy as well as from oxidative stress that originated within the microenvironment. This observation suggests that upon cancer chemotherapy, anticancer drugs strongly attack the healthy cells in the microenvironment rather than the cancer cells, which makes the chemotherapy less effective. To counteract this activity, the combined use of NRF2 inducers with anticancer drugs may be useful to overcome this limitation of conventional cancer chemotherapy.

Oltipraz (4-methyl-5-(2-pyrazinyl)-3H-1,2-dithiole-3-thione) is a schestosomicide that is, and also a well-known NRF2 inducer. Oltipraz inhibits the formation of various cancers in rodent models (44, 91). The clinical cancer chemoprevention trials of this drug have progressed (92, 93). Whereas Oltipraz attenuates the progression of non-alcoholic steatohepatitis (NASH) and is now in phase 3 clinical trial against NASH in South Korea, Oltipraz has not yet been developed as an anticancer drug.

There are some NRF2 inducers in dietary supplements. Sulforaphane from broccoli sprouts (69) induces cytoprotective enzymes through direct binding to C151 of KEAP1 (94). It has been shown that curcumin from turmeric activates NRF2 through the inhibition of KEAP1 (70); however, it remains unclear whether curcumin reacts with cysteine residues of KEAP1. Carnosic acid from rosemary activates NRF2 by directly binding to cysteine residues in KEAP1 (71). To demonstrate the protective effect of broccoli sprouts containing precursors of sulforaphane against carcinogens such as aflatoxin and airborne toxicants, a clinical trial using broccoli sprouts was performed in China (95). Individuals who received a broccoli sprout-based beverage exhibited reduced toxic metabolite levels and increased detoxified metabolite levels.

In addition to these chemo-preventive uses of NRF2 inducers, there may be alternative uses of NRF2 inducers in the chemo- and radio-therapy of cancer patients. NRF2-addicted cancers are resistant to these cancer therapies due to their high intrinsic NRF2 activity. Therefore, anticancer drugs attack micro-environmental cells or immune cells more severely than the cancer cells, which already express a high level of NRF2 and cytoprotective enzymes. To this end, the combined use of an NRF2 inducer with conventional anticancer agents may better protect the host cells and improve the efficacy of anticancer drugs against NRF2-addicted cancers.

## Conclusion

Both NRF2 inducers and NRF2 inhibitors are expected to function as anticancer drugs. However, the targets of these drugs are significantly different: NRF2 inducers act to protect normal cells from carcinogens, whereas NRF2 inhibitors act to suppress the proliferation of cancer cells that have acquired aberrant NRF2 activation or NRF2 addiction. However, many questions related to the NRF2 inducers and inhibitors remain and must be resolved before the NRF2 inducers and inhibitors can be judiciously applied in anticancer therapy. For example, methods to assess the NRF2 addiction status of each cancer need to be established. Notably, in human HCC biopsies, *G6PD* and *NQO1* mRNAs can be used as markers that correlate well with metastatic status and poor prognosis (96). These markers could also be used as accurate indicators of NRF2 activity or NRF2 addiction. In addition, an emerging possibility is to use NRF2 inducers for cancer chemotherapy in combination with conventional anticancer agents or even NRF2 inhibitors. In this regard, there is a concern whether long-term application of NRF2 inducers may eventually support the growth of cryptic cancer-initiating cells into real cancer cells. While available lines of evidence from us and other laboratories do not support this concern, further investigations are needed to exclude this possibility.

## Author Contributions

Both authors equally contribute to the description.

## Conflict of Interest Statement

The authors declare that the research was conducted in the absence of any commercial or financial relationships that could be construed as a potential conflict of interest.
